# Aerodynamic evaluation of surgical design for the stenosis correction of airway

**DOI:** 10.3389/fcvm.2024.1359844

**Published:** 2024-03-28

**Authors:** Wenjie Bao, Andi Liao, Pingping Yu, Juanya Shen, Wenjing Zhao, Lifeng Ding

**Affiliations:** ^1^ Department of Scientific Management, Shanghai Children’s Medical Center, School of Medicine, Shanghai Jiao Tong University, Shanghai, China; ^2^ Shanghai Engineering Research Center of Virtual Reality of Structural Heart Disease, Shanghai Children’s Medical Center, School of Medicine, Shanghai Jiao Tong University, Shanghai, China; ^3^ Institute of Pediatric Translational Medicine, Shanghai Children’s Medical Center, School of Medicine, Shanghai Jiao Tong University, Shanghai, China; ^4^ Department of Cardiothoracic Surgery, Shanghai Children’s Medical Center, School of Medicine, Shanghai Jiao Tong University, Shanghai, China; ^5^ Shanghai Institute for Pediatric Congenital Heart Disease, Shanghai Children’s Medical Center, School of Medicine, Shanghai Jiao Tong University, Shanghai, China; ^6^ China-UK Low Carbon College, Shanghai Jiao Tong University, Shanghai, China; ^7^ Key Laboratory for Power Machinery and Engineering, Ministry of Education, Shanghai Jiao Tong University, Shanghai, China; ^8^ College of Mechanical Engineering, Zhejiang University of Technology, Hangzhou, Jiangsu, China; ^9^ Department of Traditional Chinese Medicine, Shanghai Children’s Medical Center, School of Medicine, Shanghai Jiao Tong University, Shanghai, China

**Keywords:** congenital tracheal stenosis, obstructive sleep apnea syndrome, aerodynamics, virtual surgery, simulation

## Abstract

**Introduction:**

Congenital tracheal stenosis (CTS) is a rare but life-threatening disease that can lead to respiratory dysfunction in children. Obstructive sleep apnea syndrome (OSAS) in children is characterized by prolonged partial upper airway obstruction and/or intermittent complete obstruction. Both of the diseases require surgical intervention. Although respective treatments of these two diseases are clear, there is a lack of literature discussing the surgical treatment of patients with CTS complicated by OSAS.

**Methods:**

We conducted a patient-specific study of patient with CTS complicated by OSAS. Computer-aided design was used to simulate surgical correction under different surgical sequences. Computational fluid dynamics was used to compare the outcomes of different sequences.

**Results:**

Aerodynamic parameters, pressure drop, velocity streamlines, wall shear stress (WSS), and the ratio of airflow distribution and energy loss rate were evaluated. An obvious interaction was found between the two diseases in different surgical sequences. The order of correction for CTS or OSAS greatly affected the aerodynamic parameters and turbulence flows downstream of tracheal stenosis and upstream of epiglottis. The CTS and OSAS had mutual influences on each other on the aerodynamic parameters, such as pressure drops and WSS.

**Discussion:**

When evaluating the priority of surgical urgency of CTS and OSAS, surgeons need to pay attention to the state of both CTS and OSAS and the physiological conditions of patients. The aerodynamic performance of the uneven airflow distribution and the potential impact caused by the correction of CTS should be considered in surgical planning and clinical management.

## 1 Introduction

Congenital tracheal stenosis (CTS) is a rare but life-threatening disease that can lead to respiratory dysfunction in children. It is mainly characterized by the symptoms of stridor, wheezing, apnea, and recurrent upper respiratory tract infections or pneumonia. The severity of the symptoms lies on the length of the affected trachea, the diameter of the narrowing lumen, the presence of concurrent malformations, and so on. Tracheal reconstruction surgery is regarded as the best treatment of severe tracheal stenosis, with surgical techniques including tracheal resection with end-to-end anastomosis, slide tracheoplasty, and patch tracheoplasty (1–5). Obstructive sleep apnea syndrome (OSAS) in children is a “disorder of breathing during sleep characterized by prolonged partial upper airway obstruction and/or intermittent complete obstruction (obstructive apnea) that disrupts normal ventilation during sleep and normal sleep patterns” (6). The first-line treatment for OSAS is tonsillectomy with or without an adenoidectomy, also known as an adenotonsillectomy (AT) (7). Although the respective treatments for these two diseases are clear, there are few clinical cases presented as CTS complicated by OSAS. There is a lack of literature discussing patients with CTS complicated by OSAS. The upper airway restriction caused by OSAS and the respiratory dysfunction caused by CTS may result in different aerodynamic changes, which can influence each other and thus aggravate the symptoms.

With the development of medical imaging and computational fluid dynamics (CFD), we have gained a better understanding of CTS and OSAS at a three-dimensional (3D) level by evaluating aerodynamic properties. To conduct a CFD analysis of the airflow in those abnormal airways, we constructed computational 3D models according to the individual images and simulated the airway flows. Multiple data were provided through the analysis, such as pressure drop, wall shear stress (WSS), and energy loss rate (ELR), which can be hard to measure directly in clinical practice. In recent years, a growing number of studies have been conducted using CFD to analyze aerodynamic changes (8–14). Morita et al. measured the energy flux and the minimum cross-sectional area of the trachea (MCAT) of the patients with CTS before and after surgery using CFD (8). The energy flux correlated positively with clinical respiratory status before and after surgery, suggesting that CFD can be an additional evaluation tool for recognizing the respiratory status of CTS. Mimouni-Benabu et al. constructed 3D geometries of the trachea in eight children with CTS (10). They determined flow velocity, static and total airway pressure, and pressure drop across the entire trachea, which enabled a classification based on the severity of stenosis. Zhu et al. utilized CFD to study minimum cross-sectional area, pressure drop, and velocity in patients with OSAS after H-uvulopalatopharyngoplasty (11). All the studies mentioned above indicate that CFD is an appropriate method of studying the aerodynamics in both CTS and OSAS in patient-specific studies. It is a viable way of evaluating the aerodynamic interplay in patients with CTS complicated by OSAS, consequently aiding in the clinical selection of surgical sequence and timing.

**Figure 1:**
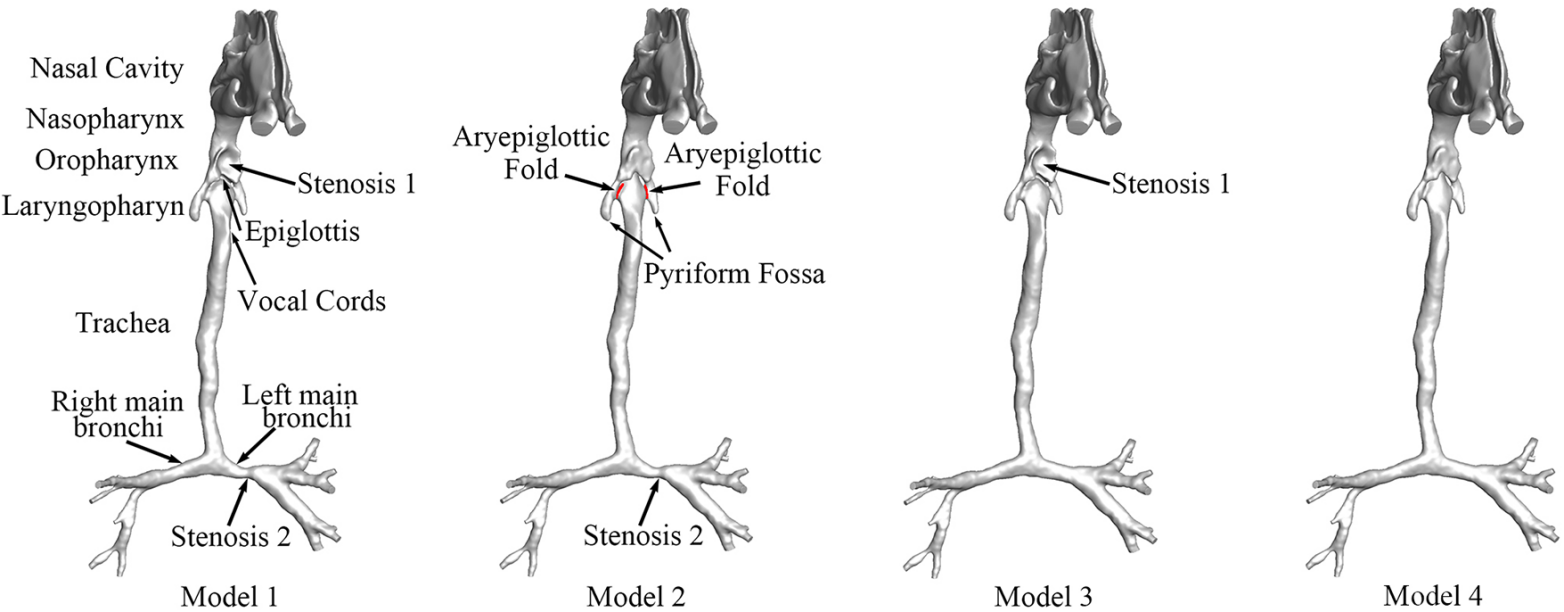
The anatomical models of airway before and after surgery. Stenosis 1: the stenosis at the oropharynx caused by compression of the enlarged adenoid, namely, OSAS. Stenosis 2: CTS at left main bronchi. Model 1: preoperative model with OSAS and CTS. Model 2: postoperative model with OSAS corrected. Model 3: postoperative model with CTS corrected. Model 4: postoperative model without OSAS and CTS.

In the present study, we constructed a patient-specific 3D model with both CTS and OSAS on the basis of medical imaging. Computer-aided design (CAD) was used to construct new models by correcting CTS or OSAS. The OSAS was corrected in Model 2, and the CTS was corrected in Model 3. Model 4 was created as the normal control with both CTS and OSAS corrected virtually. The anatomical airway models before and after surgery are shown in Figure 1. We evaluated the aerodynamic characteristics under different surgery sequences by calculating pressure drop, velocity streamlines, WSS, airway distribution ration, and energy loss rate, and both inspiration and expiration were included. The aim of this study was not only to estimate the aerodynamic features of patients with CTS complicated by OSAS, but also to conduct virtual surgery that correct CTS and OSAS in different orders to choose a better surgery sequence.

## 2 Methods

### 2.1 Clinical data acquisition

Approval was obtained from the local institutional review board and regional research ethics committee of Shanghai Children's Medical Center (SCMC) Affiliated to Shanghai Jiao Tong University School of Medicine before the studies were carried out. Written informed consent for the participation and publication of the present study was obtained from the patient's parents.

The patient diagnosed with CTS complicated by OSAS was included; the disease manifested as inspiratory stridor with wheezing and apnea. The preoperative computed tomography (CT) data of the patient were collected using a 64-slice spiral CT machine (Discovery HD750; GE Healthcare, Waukesha, WI, USA) with a slice thickness of 0.625 mm and resolution of 512 × 512 pixels. This was stored in a digital imaging and communications in medicine (DICOM) format. Adenoid hypertrophy was found in the nasopharyngeal endoscopy, which occluded two-thirds of the posterior nostrils.

### 2.2 Generation of 3D airway models

The patient-specific CT data were used by the medical image software Materialise-Mimics Innovation Suite 21.0 (Materialise NV, Leuven, Belgium) to generate the 3D airway model. Figure 1 shows the geometry of the total airway after surface smoothing with the main bronchus. The 3D airway model (Model 1) displayed severe stenosis at the oropharynx and left main bronchi. The model was stored in a stereo-lithography interface (STL) format to be used with CAD software to perform virtual surgeries.

The virtual surgery was carried out to simulate the three possible schemes of the stenosis corrections, stenosis 1 and stenosis 2. We applied the CAD software Materialise-3-matic 13.0 (Materialise NV, Leuven, Belgium) to rebuild the original stenotic segment to normal size based on the analysis of the patient-specific CT images. Three virtual models, Models 2, 3, and 4, were generated, as shown in Figure 1.

### 2.3 CFD analysis

#### 2.3.1 Governing equation

Previous studies showed that the airflow can be assumed as incompressible in the presence of turbulence in the airway (8, 11, 15–17). Thus, the airflow was assumed to be incompressible in this study. The motion of the air flowing into the airway was described using the following 3D incompressible Navier–Stokes (N-S) equations:

(1) $$\{\begin{array}{c} \frac{\partial }{\partial t}(\rho u_{i})+\frac{\partial }{\partial x_{j}}(\rho u_{i}u_{j})=-\frac{\partial p}{\partial x_{i}}+\frac{\partial }{\partial x_{j}}\left[\mu \left(\frac{\partial u_{i}}{\partial x_{j}}+\frac{\partial u_{j}}{\partial x_{i}}\right)\right]+f_{i}\\ \frac{\partial p}{\partial t}+\frac{\partial }{\partial x_{j}}(\rho u_{j})=0 \end{array}$$

where *u* is the velocity components *u_i,j,k_* (*i, j, k* = 1, 2, 3) in *x*, *y*, and *z* directions, *ρ* is the air density, *μ* is the air viscosity, *p* is the pressure, and *t* is the time. The term *f_i_* expresses the action of body forces. We assumed the density and the viscosity of the airflow were constant with the value of 1.161 kg/m^3^ and 1.864 ×10^−5^ kg/(m s), respectively (10, 18).

The Reynolds number (*Re*) was calculated. The transition to turbulence usually occurs at a local *Re* of approximately 2,000 (19). Owing to the complexity of the nasal airway, the transition to turbulence may take place at a lower *Re* (19). In our study, the *Re* reached 1,868 at the carina and the low Reynolds number (LRN) *k*–*ω* model was adopted (20). It was a commonly used two-equation eddy viscosity model based on the equations for *k* (turbulent kinetic energy) and *ω* (specific turbulent dissipation rate) defined below.

*k* equation:

(2) $$\bar{u_{j}}\frac{\partial k}{\partial x_{j}}=\tau_{ij}\frac{\partial \bar{u_{i}}}{\partial x_{j}}-\beta^{*}k\omega +\frac{\partial }{\partial x_{j}}\left[(v+\sigma_{k}v_{T})\frac{\partial k}{\partial x_{j}}\right]$$

*ω* equation:

(3) $$\bar{u_{j}}\frac{\partial \omega }{\partial x_{j}}=\alpha \frac{\omega }{k}\tau_{ij}\frac{\partial \bar{u_{i}}}{\partial x_{j}}-\beta \omega^{2}+\frac{\partial }{\partial x_{j}}\left[(v+\sigma_{\omega }v_{T})\frac{\partial \omega }{\partial x_{j}}\right]$$

*ν*, *ν_T_*, and *τ_ij_* are kinetic molecular viscosity, turbulent viscosity, and Reynolds stress tensor, respectively, while *ν_T_ = C_µ_f_µ_k/ω* and function.

(4) $$f_{\mu }=\exp[-3.4/{\left(I+R_{T}/50\right)}^{2}]$$

with *R_T_ = k/µω*, *µ* being dynamic molecular viscosity. Model constants are *C_µ_* = 0.09, *α* = 0.555, *β* = 0.8333, β* = 1, and *σ_k_* =* σ_w_* = 0.5.

WSS indicates the complex interaction between airflow and the wall of the airway.

(5) $$\tau_{wall}={-\mu \frac{\partial u_{x}}{\partial_{y}}|}_{y=0}$$

where *μ* is the viscosity, *u_x_* is the velocity of the air near the airway wall, and *y* is the height above the airway wall.

Air distribution ratio (ADR) was defined as the percentage of the airflow into the left main bronchi divided by the total air inflow, calculated as the following equation:

(6) $$\mathrm{ADR}=\frac{Q_{LMB}}{Q_{Total}}\times 100\text{\%}$$

where *Q_LMB_* represents the volumetric airflow into the left main bronchi and *Q_Total_* represents the total airflow into the airway.

Energy loss (EL) indicates the energy difference between the inlets and the outlets of the airway flow domain, and energy loss rate (ELR) was defined as the percentage of EL decrease relative to the EL of Model 1, which reflects the energy dissipation and the load of breath of different surgical designs. The ELR can be calculated by equation (7),

(7) $$\mathrm{ELR}=\frac{\Delta EL}{EL_{1}}\times 100\text{\%}=\frac{\left|EL_{n}-EL_{1}\right|}{EL_{1}}\times 100\text{\%}$$

where *n* = 2, 3, 4, *EL_1_* represents the energy loss of Model 1, and *EL_n_* represents the energy loss of Model *n*.

#### 2.3.2 Mesh generation

The mixed grids were generated for the calculation by the commercial software ANSYS®-ICEM CFD 2020 (ANSYS Inc., USA). The five boundary-fitted prism layers were created at the near-wall regions of the airway for each model and the tetrahedral grids were used to cover the interior domains of the airway. Grid-independent verification was carried out to find appropriate grids for the calculation. We learned that when the grid number of each model was above 3.0 million, it was more efficient to obtain accurate results in a relatively short time. Table 1 lists the details of the grid information in the calculation.

**Table 1:** Information of each model mesh.

	Model 1	Model 2	Model 3	Model 4
Total elements	3,277,527	3,280,925	3,283,435	3,287,911
Total nodes	58,987	59,0633	59,1017	59,1850

#### 2.3.3 Boundary conditions

The pre-treatment state and three possible post-treatment states were simulated. The boundary conditions of the four states were derived from the average and maximum mass flow rates during inspiration and expiration from the patient’s pulmonary function test. The mean and maximum flow rate of the inspiration phase were 73 and 106 mL/s, and those of the expiratory phase were 60 and 85.5 mL/s. During the inspiratory phase, the inlets were the two nostrils with the average and maximum flow rate of the inspiration phase separately applied; the outlets were at the end of trachea. The outlet pressures were assumed to meet the condition of the atmospheric pressure in the two front nostrils. The airflow direction was opposite in the expiratory phase; the inlets were at the end of trachea and the outlets were the two nostrils. The average and maximum flow rate of the expiratory phase, −60 and −85.5 mL/s, respectively, were applied in the outlets. The pressures at the inlets were assumed to meet the atmospheric pressure conditions in the two front nostrils.

#### 2.3.4 Calculation

The software ANSYS®-Fluent 2020 (ANSYS Inc.) was used for the simulation of the complex airflow. The Semi-Implicit Method (SIMPLE) and the Second Order Upwind scheme were employed for the discretization of the incompressible N-S equations (equation (1)) and the standard *k–ω* (equation (2–4)) turbulence model. The time step was 10^−4^ s and the convergence criterion was 10^−5^ for each time step. The post-processing analysis of the calculation results includes the pressure drop, velocity streamlines, WSS (equation (5)), ADR (equation (6)), and ELR (equation (7)).

## 3 Results

### 3.1 Pressure

**Figure 2:**
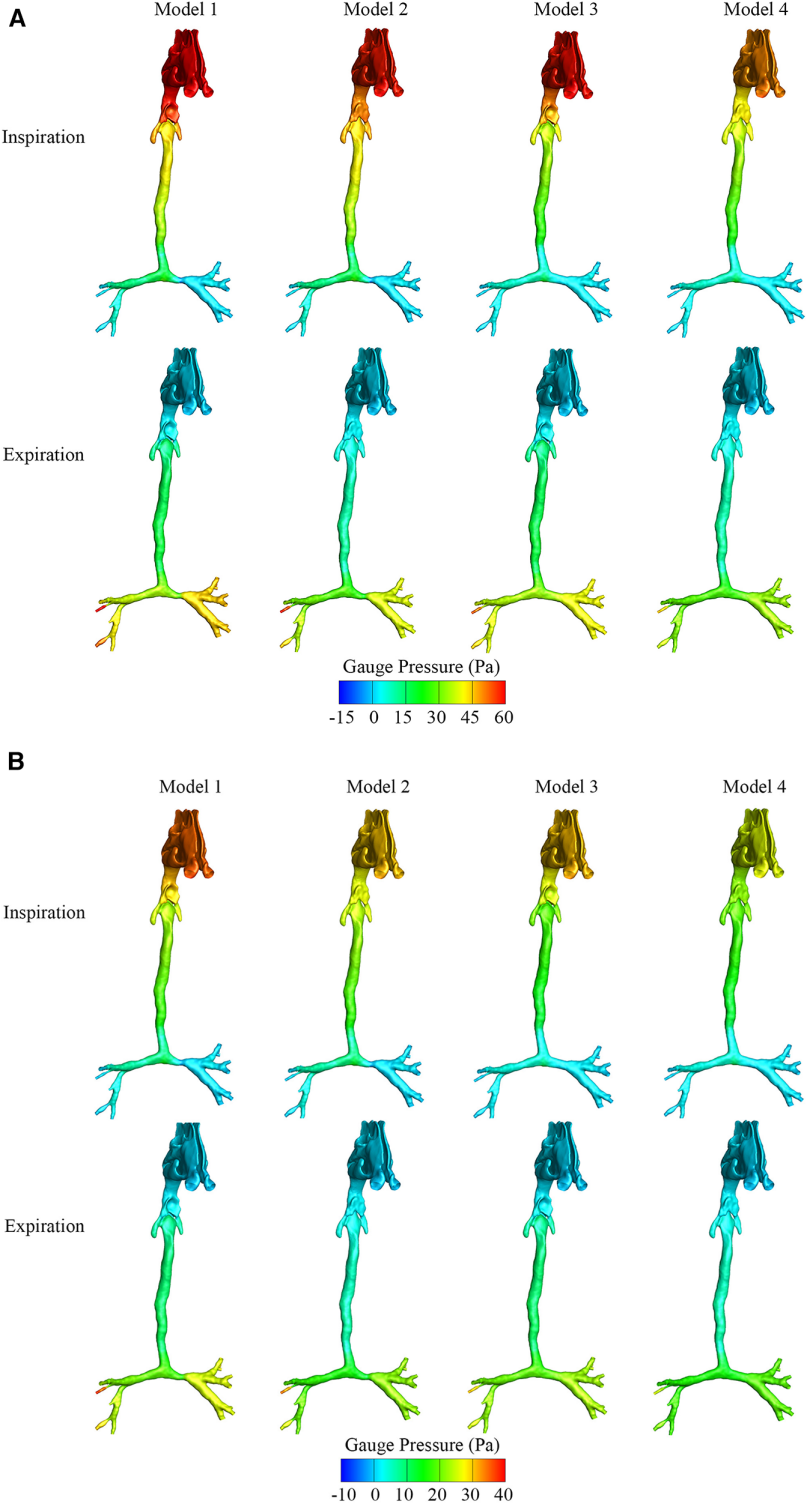
(**A**) The maximum gauge pressure of four airway models in the inspiratory and expiratory phase. (**B**) The average gauge pressure of four airway models in the inspiratory and expiratory phase.

Figures 2A,B show the maximum and average gauge pressure distribution of the four models in the inspiratory and expiratory phases. There was an obvious increase of gauge pressure above the oropharynx in Models 1–3 during the inspiratory phase, but there was a small change in the expiratory phase. As shown in Figure 2A, the maximum pressure drop around the oropharynx of the model with both CTS and OSAS was approximately 18 Pa during inspiration in contrast to 5 Pa during expiration. In Figure 2B, the average pressure drop around the oropharynx of the model with both CTS and OSAS was approximately 7 Pa during inspiration in contrast to 3 Pa during expiration. It indicated that the obstruction of the upper airway caused by both CTS and OSAS affected inspiration more than expiration. During inspiration, the maximum gauge pressure at the epiglottic cyst was approximately 55 Pa in Model 1, 47 Pa in Model 2, 43 Pa in Model 3, and 39 Pa in Model 4, the average gauge pressure at the epiglottic cyst was approximately 27 Pa, 25 Pa, 24 Pa, and 18 Pa in Models 1, 2, 3, and 4, respectively. Comparing the gauge pressure distribution in the CTS and OSAS models with the model of both diseases in the inspiratory phase, the gauge pressure increased obviously at the position of the epiglottic cyst and upstream of the epiglottis after combining CTS with OSAS. In addition, the gauge pressure of the epiglottic cyst was higher in Model 2 than in Model 3, and both were higher than in Model 4. In the expiratory phase between these models, there was a slight change in gauge pressure distribution at the laryngopharynx and trachea, but no significant change at the oropharynx. It manifested that the correction of CTS can lower the gauge pressure at the epiglottic cyst of OSAS during inspiration. But correcting OSAS cannot bring the gauge pressure to normal at the epiglottic cyst, indicating that the correction of CTS affected the gauge pressure at the epiglottic cyst of OSAS. The highest gauge pressure was found at the epiglottic cyst of OSAS in Model 1, which was more significant during inspiration.

### 3.2 Velocity streamlines

**Figure 3:**
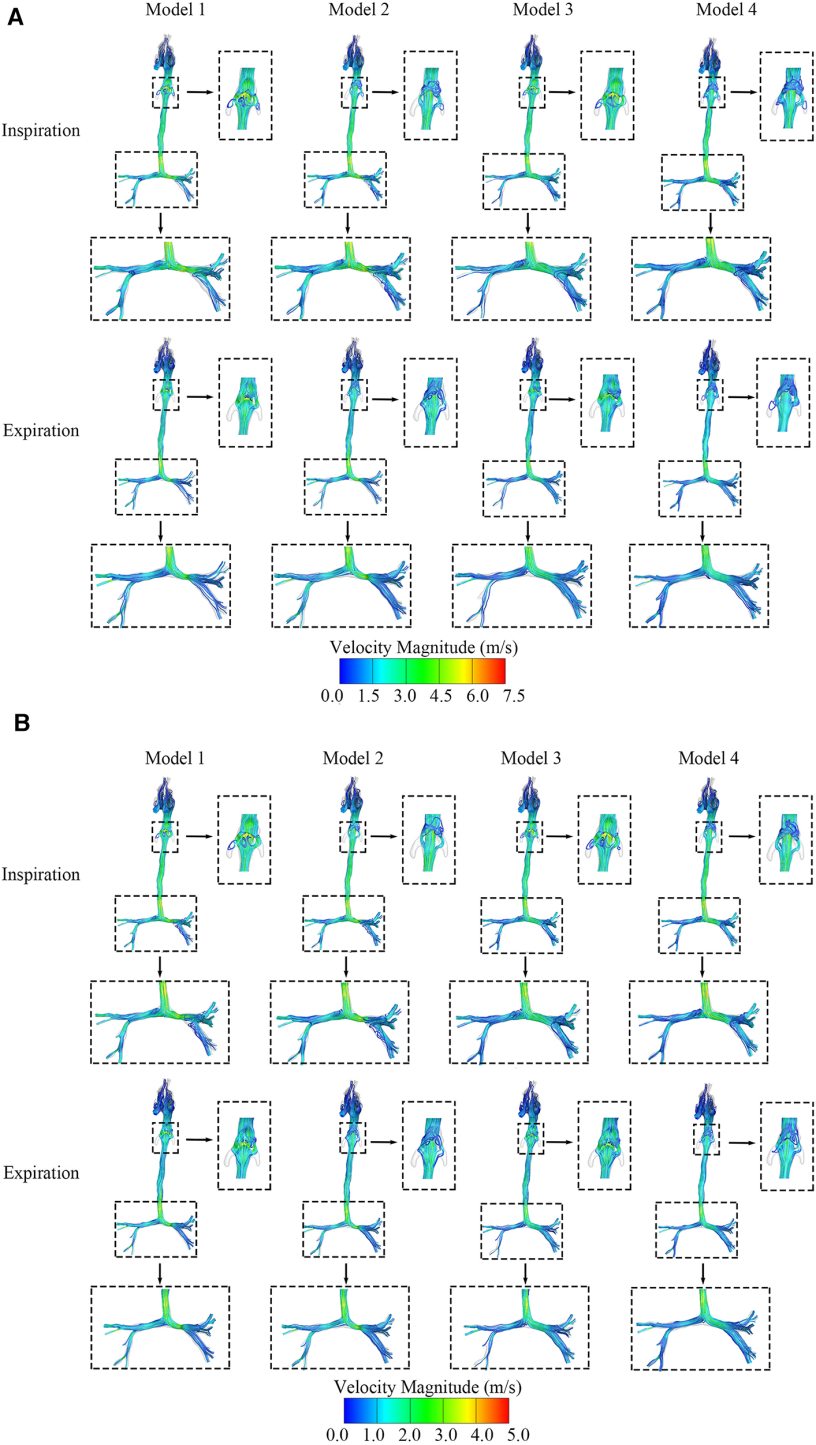
(**A**) The maximum velocity streamlines distribution of four airway models in the inspiratory and expiratory phase. (**B**) The average velocity streamlines distribution of four airway models in the inspiratory and expiratory phase.

Figures 3A,B show the maximum and average velocity magnitude of the four models during both inspiration and expiration. Turbulence flows occurred at the downstream regions of the segments of tracheal stenosis and the regions of the epiglottic cyst. The average velocity magnitude during inspiration was approximately 3.1 m/s at the epiglottis and 3.4 m/s at the left main bronchus stenosis in Model 1 with both CTS and OSAS. The average velocity magnitude was approximately 3.5 m/s at the left main bronchus stenosis in Model 2 and 3.1 m/s at the epiglottis in Model 3.

### 3.3 Wall shear stress

**Figure 4:**
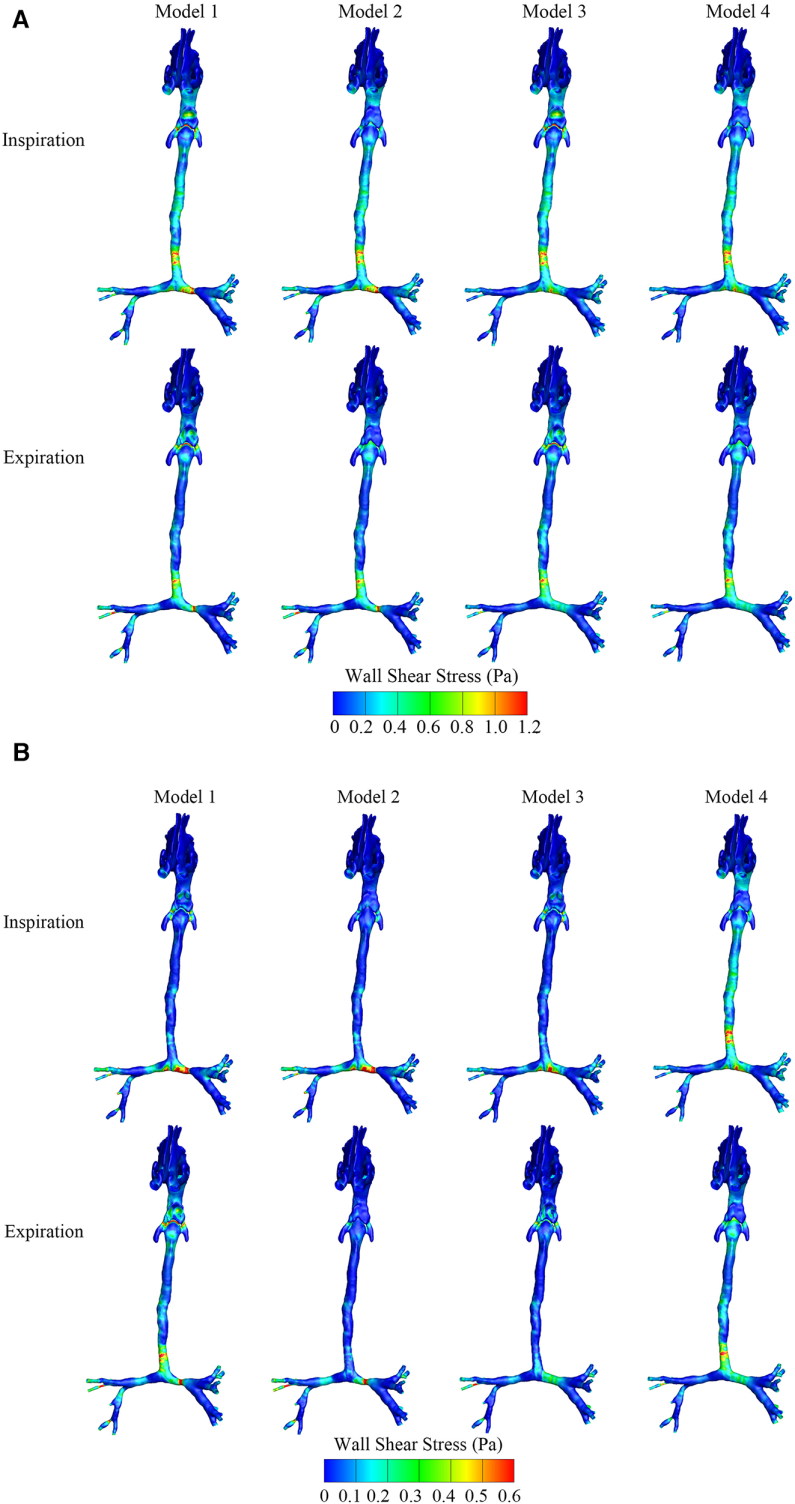
(**A**) The maximum wall shear stress distribution of four airway models in the inspiratory and expiratory phase. (**B**) The average wall shear stress distribution of four airway models in the inspiratory and expiratory phase.

As shown in Figures 4A,B, the maximum and average WSS of the four models in the inspiratory and expiratory phases were calculated. During both the inspiratory and expiratory phases, the high WSS region occurred at the level of the epiglottis in the OSAS model and the tracheal stenosis in the CTS model, both in the airway obstruction region. Comparing the WSS of the CTS model combined with OSAS to the CTS and OSAS models, respectively, in the expiratory phase, the average WSS at the level of both the epiglottis and tracheal stenosis were higher than that when the CTS and OSAS existed alone. At the position of the epiglottis, the WSS was 0.58 Pa in Model 1, 0.02 Pa in Model 2, 0.14 Pa in Model 3, and 0.24 Pa in Model 4. At the position of tracheal stenosis, the WSS was 0.59 Pa in Model 1, 0.58 Pa in Model 2, 0.25 Pa in Model 3, and 0.25 Pa in Model 4. However, there were no significant differences between them in the inspiratory phase. It was indicated that the CTS and OSAS have mutual influences on each other on the distribution of WSS, which was more significant during expiration.

### 3.4 Airflow distribution ratio

**Figure 5:**
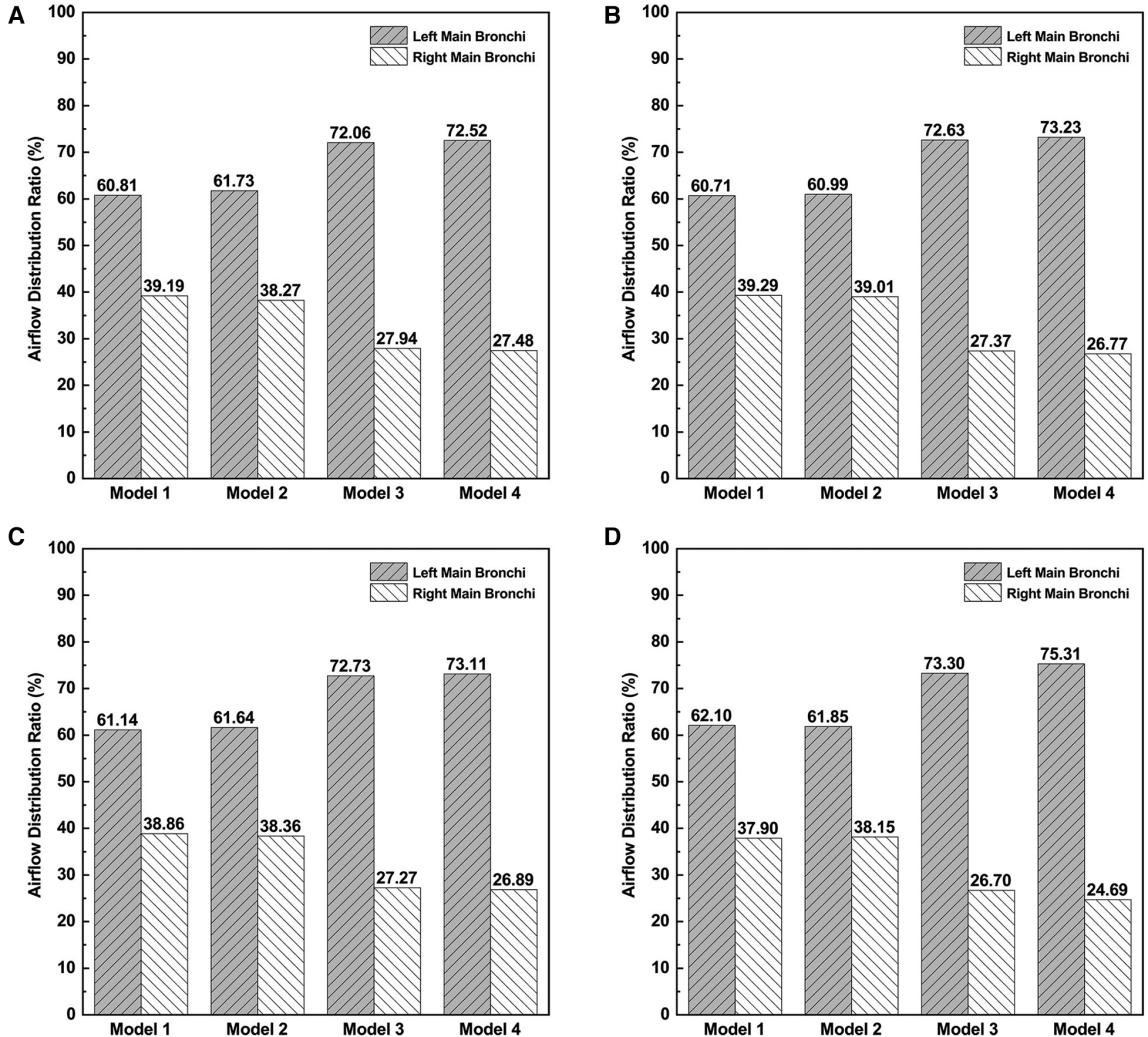
The airflow distribution ratios to right main bronchi and left main bronchi. (**A**) The airflow distribution ratio at the inspiratory peak. (**B**) The airflow distribution ratio at the expiratory peak. (**C**) The average airflow distribution ratio during inspiration. (**D**) The average airflow distribution ratio during expiration.

The ADR to the right and left main bronchi was calculated and compared in Figure 5. The ADR to the right and left main bronchi was calculated and compared in Figure 5. The left/right airflow distribution ratios were very similar among Models 1 and 2, close to 6:4, while the left/right airflow distribution ratios were approximately 7:3 in Models 3 and 4. The correction of CTS greatly increased the airflow to the left main bronchus. The correction of OSAS had no significant effects on airflow distribution. These results indicated that the altered ADR in patients with CTS combined with OSAS mainly depended on the CTS instead of the OSAS. The correction of CTS aggravated the uneven distribution of the airflow between the left and right main bronchi.

### 3.5 Energy loss rate

Tables 2, 3 describe the maximum and average ELR during inspiration and expiration, respectively. To compare the changes in EL after surgery, the degree of EL decrease was evaluated and defined as the percentage of EL decrease relative to the EL of Model 1, listed in Tables 2, 3. The maximum EL decreased by 14.12%, 11.80%, and 25.51% in Models 2, 3, and 4, respectively, at the inspiratory peak. Compared with Model 1, the average EL during the inspiratory phase decreased by 13.62%, 11.71%, and 25.66% in Models 2, 3, and 4, respectively. The percentage of EL decrease in Model 4 was approximately equal to the sum of that of Models 2 and 3. This meant that the model with both OSAS and CTS alleviated is the best performing in controlling EL. It is also indicated that the degree of EL decrease varied after alleviating CTS or OSAS only. Corrected OSAS was associated with a better EL performance in controlling EL compared to the corrected CTS.

**Table 2:** The percentage of the maximum energy loss decrease after surgery.

	Model 2	Model 3	Model 4
Inspiration (%)	14.12	11.80	25.51
Expiration (%)	13.98	10.96	24.78

**Table 3:** The percentage of the average energy loss decrease after surgery.

	Model 2	Model 3	Model 4
Inspiration (%)	13.62	11.71	25.66
Expiration (%)	13.91	11.01	24.73

## 4 Discussion

For patients with CTS complicated by OSAS, the symptoms depend on both upper airway restriction caused by OSAS and the respiratory dysfunction caused by CTS. The complex interplay of CTS and OSAS may result in different aerodynamic changes, thus aggravating the symptoms. Surgery is the main treatment for relieving the symptoms of airway obstruction. Meanwhile, a serious surgical plan including the options for different surgery sequences plays an important role in improving the success rate and postoperative recovery. Common clinical examinations, such as CT and endoscopy, enable the identification of the presence of CTS and OSAS. However, it is hard to acquire the aerodynamic characteristics of the airway, giving little aid to the preoperative surgery design and the prediction of surgery efficacy and postoperative recovery. In the present study, a patient-specific 3D model with both CTS and OSAS was constructed, and three possible postoperative models with different surgery sequences were simulated through virtual surgery assisted by CAD. CFD simulations were also used to evaluate the differences of the aerodynamic characteristics under different surgery sequences. We also calculated the aerodynamic parameters, such as pressure drop, velocity streamlines, WSS, ELR, and the ratio of airflow distribution, which cannot be acquired from common clinical examinations, to evaluate the local aerodynamic features of the stenosis.

As is shown in the results, owing to the constriction of the airway at the epiglottis and left main bronchi, the airflow manifested as an obvious fluctuation and high-velocity regions. In the results, the maximum gauge pressure upstream of the epiglottis of the CTS combined with OSAS model during inspiration was much higher than either CTS or OSAS on their own. However, there is no obvious influence on that during expiration while combining CTS with OSAS. This may be due to the different maximum flow rates of inhalation and exhalation. However, the difference in maximum flow rates does indeed reflect the physiological condition of the patient. Therefore, when evaluating the aerodynamic performance of patients, attention should be paid to the physiological condition of patients. Similarly, the ELR during both inspiration and expiration of the CTS combined with OSAS model was obviously higher than if those two diseases existed alone. The results showed that the aerodynamic features of CTS combined with OSAS are not simply the sum of the characteristics of each of the two diseases and there exists an interplay of the aerodynamic changes between the two diseases. WSS was considered to be the embodiment of the interplay between the airflow and the tracheal wall. The WSS of the model with both CTS and OSAS shows a greater increase during the expiratory phase than the inspiratory phase, which indicated that there is a greater interaction between the airflow and the tracheal wall during the expiratory phase. The altered left/right airflow distribution ratio in patient with CTS combined with OSAS is close to 6:4, which mainly depends on the CTS instead of OSAS. The correction of CTS increases the airflow distribution of the left and right main bronchi, which is close to 7:3, but aggravates the uneven distribution of the airflow of the left and right main bronchi to some extent. Thus, when choosing surgery sequences for the disease, surgeons need to pay attention to the state of both CTS and OSAS and the physiological condition of patients when evaluating the aerodynamic performance, and then evaluate the surgical urgency of CTS and OSAS.

The present study has some limitations. First, we assumed the airway wall as rigid and the airflow in the upper airway as incompressible and Newtonian flow. Although the most part of the airway wall in patients with CTS is characterized by complete tracheal cartilage rings, there still exists the mucosal layer on the tracheal cartilage. In addition, the airflow and the soft tissues in the pharyngeal wall interplay in a complex way, which makes it challenging to incorporate all these factors into modeling. Considering the situation, further studies need to be carried out. Second, we used a fixed airway model based on CT images of inspiration to simulate both inspiration and expiration. In addition, OSAS existing as an intermittent obstruction varied with the respiratory cycle. However, the differences in the anatomic structures between the expiratory and inspiratory phases were not significant for the pediatric patient included in this study. The modeling error caused by the use of CT images during inspiration was considered to be acceptable. Third, the sample size was small. The present study only included one specific patient with CTS complicated by OSAS. However, this study based on a specific patient still has significance because of the fitted boundary conditions and suitable calculation settings. A larger sample size of patients with CTS complicated by OSAS needs to be analyzed in future research. Fourth, we rectified the CTS and OSAS virtually without considering a specific surgery method, which may have an impact on the results of the aerodynamic features.

## 5 Conclusion

An obvious interaction was found between the two diseases during different surgical sequences. The order in which to correct the stenosis of CTS or OSAS greatly affected the aerodynamic parameters and turbulence flows downstream of tracheal stenosis and upstream of the epiglottis. The CTS and OSAS had mutual influences on each other on the aerodynamic parameters, such as pressure drops and WSS. When evaluating the surgical urgency of CTS and OSAS, surgeons need to pay attention to the state of both CTS and OSAS and the physiological condition of patients. The aerodynamic performance of the uneven airflow distribution and the potential impact caused by the correction of CTS should be considered in surgical planning and clinical management. CFD facilitates the evaluation of the aerodynamic characteristics by calculating pressure drops, WSS, airflow distribution, and ELR, assisting the surgery design and the prediction of surgery efficacy. According to the abovementioned statements, it is very promising to apply CFD into clinical practice widely.

## Data Availability

The original contributions presented in the study are included in the article/Supplementary Material, further inquiries can be directed to the corresponding authors.
